# Isolation and Characterization of Polymorphic Microsatellite Loci from *Metapenaeopsis barbata* Using PCR-Based Isolation of Microsatellite Arrays (PIMA)

**DOI:** 10.3390/ijms13032763

**Published:** 2012-03-01

**Authors:** Tzen-Yuh Chiang, Tzong-Der Tzeng, Hung-Du Lin, Ching-Ju Cho, Feng-Jiau Lin

**Affiliations:** 1 Department of Life Sciences, National Cheng Kung University, Tainan 701, Taiwan; E-Mails: tychiang@mail.ncku.edu.tw (T.-Y.C.); lovego8@hotmail.com (C.-J.C.); 2 Department of Leisure, Recreation and Tourism Management, Shu-Te University, Kaohsiung 824, Taiwan; E-Mail: tdtzeng@stu.edu.tw; 3 The Affiliated School of National Tainan First Senior High School, Tainan 701, Taiwan; E-Mail:varicorhinus@hotmail.com

**Keywords:** microsatellite, management, PIMA, RAPD-PCR enrichment, *Metapenaeopsis barbata*

## Abstract

The red-spot prawn, *Metapenaeopsis barbata*, is a commercially important, widely distributed demersal species in the Indo-West Pacific Ocean. Overfishing has made its populations decline in the past decade. To study conservation genetics, eight polymorphic microsatellite loci were isolated. Genetic characteristics of the SSR (simple sequence repeat) fingerprints were estimated in 61 individuals from adjacent seas of Taiwan and China. The number of alleles, ranging from 2 to 4, as well as observed and expected heterozygosities in populations, ranging from 0.048 to 0.538, and 0.048 and 0.654, respectively, were detected. No deviation from Hardy–Weinberg expectations was detected at either locus. No significant linkage disequilibrium was detected in locus pairs. The polymorphic microsatellite loci will be useful for investigations of the genetic variation, population structure, and conservation genetics of this species.

## 1. Introduction

The red-spot prawn, *Metapenaeopsis barbata*, is a widely distributed demersal species in Indo-West Pacific from the Gulf of Bengal to Japan and Indonesia [1–3]. It is one of the abundant prawns with high commercial values [2,4]. It was overfished from 1995 to 1996 around Taiwan [5]. Furthermore, the populations of this species declined in the past decade likely due to the effective benthic trawling [6] and ecological destruction. The life history, morphometric variation and fishery biology of these shrimps have been well studied [2,5,7–10]. Recently, Chu [11,12] investigated the population structure and historical demography of the whiskered velvet shrimp using one intron of elongation factor-1α gene and the mitochondrial DNA control region. For practicing conservation and understanding the genetic structuring across populations, codominant and highly polymorphic molecular markers are desired. Highly polymorphic microsatellite DNAs have been widely used in many aquacultural species to evaluate the genetic diversity [13,14], construct genetic maps [15,16], and determine species’ lineages [17], as well as for conservation and management in shrimps [16]. In order to protect and manage these overexploited wild resources of *M. barbata*, molecular markers are urgently needed to develop suitable strategies to maintain sustainable populations

## 2. Results and Discussion

In total, 61 individuals of *M. barbata*, including 21 from Taichung, Taiwan and 40 from Fujian, China, were collected. Eight di-nucleotide SSR loci were isolated. The characteristics of the innovative microsatellite loci and variability measures across two populations are described in Table 1. The number of alleles per locus ranged from 2 to 4, with an average of 3.00 in Taichung and an average 3.25 in Fujian. The observed heterozygosities (*H*_O_) and expected heterozygosities (*H*_E_) ranged from 0.048 to 0.476 (averaged at 0.327) and ranged from 0.048 to 0.667 (averaged at 0.420), respectively, in Taichung; while the *H*_O_ and *H*_E_ ranged from 0.205 to 0.538 (average = 0.331) and from 0.283 to 0.654 (average = 0.392), respectively, in Fujian. No deviation from Hardy–Weinberg expectations was detected at either locus. There was no evidence of linkage disequilibrium between any pairs of loci. The locus-wise *F*_IS_ for each population, shown in Table 2, was non-significant after Bonferroni correction [18], indicating heterozygote deficiency in all but one locus (MIMB03) in Fujian population. Microsatellite markers could be a good choice for the characterization of genetic diversity in *M. barbata* due to its reliable, informative, co-dominant nature and ease of exchange of data among different studies. The results suggest that the microsatellite DNA loci identified in this study are highly polymorphic, and that these markers can be useful for investigating the genetic structure and management of *M. barbata* populations.

## 3. Experimental Section

### 3.1. Isolation of Microsatellite Markers

In present study, we have developed eight polymorphic microsatellite markers that are specific for *M. barbata*. Genomic DNA was extracted from muscle tissues preserved in 95% ethanol by following standard phenol-chloroform procedure [19]. The enrichment of DNA fragments was carried out using the random amplified polymorphic DNA-polymerase chain reaction (RAPD-PCR) method [20] with a minor modification [21]. This PIMA (PCR isolation of microsatellite arrays) approach has been proposed [20]. It takes advantage of the fact that the RAPD fragments contain microsatellite repeats more frequently than random genomic clones [22]. Several RAPD primers were selected to amplify the target DNA fragments. The RAPD-PCR amplifications were performed in a thermal cycler (Bio-Rad) with a reaction mixture (50 μL) containing 20–100 ng DNA, 0.2 mM of each dNTP, 2 mM MgCl_2_, 0.5 U Taq polymerase (Promega), and 5 pmol of one RAPD primer. The PCR program were as follows: initial denaturing for 3 min at 95 °C for 1 cycle, 40 cycles of 1 min at 94 °C, 45 s at 42 °C, 2 min at 72 °C, followed by 10 min at 72 °C for an additional extension step. Approximately 100 ng of PCR product was ligated into a pGEM-T vector (Promega) according to the manufacturer’s instructions. The ligation mixture was then transformed into *Escherichia coli* competent cells to form the enriched microsatellite sequence library. Colonies of RAPD fragments were analyzed to verify existence of repetitive sequences. Clones were screened using repeat-specific and vector primers [20]. In positive clones, the repeat-specific and vector primers amplified DNA fragments that contain microsatellites, whereas no amplification was found in negative clones. Plasmid DNA from positives was purified using the High-Speed Plasmid Mini Kit (Geneaid). Both strands of the DNA insert were sequenced. DNA sequencing in both directions was conducted with an Applied Biosystems ABI3730 automated sequencer (Applied Biosystems). Primers for these loci were designed using PRIMER 3 software [23] and synthesized. Primers were designed according to the nucleotide sequences upstream and downstream of the repetitive DNA. A total of 8 primer pairs were designed from 8 sequences as the remaining sequences were too close to the cloning site. Polymorphisms of these microsatellite loci were assessed by 40 *M. barbata* individuals collected from Fujian Province, China and PCR conditions were optimized for each pair of primers. Reactions were performed in a total volume of 15-μL containing 10 ng of genomic DNA, 0.2 mM dNTP, 2 mM MgCl_2_, and 0.12 μM of each primer. The PCR program consisted of 94 °C for 5 min followed by 35 cycles at 94 °C for 30 s, 30 s at primer-specific annealing temperature (Table 1), 72 °C for 45 s and a final extension step at 72 °C for 5 min. Individuals were genotyped on 6% denaturing polyacrylamide gels stained with ethidium bromide straining and sized by comparison to a 10-bp DNA ladder standard (Invitrogen).

### 3.2. Data Analysis

Genotype data files were inter-converted for the various analytical software programs using CREATE [24] to minimize errors. The allele number, size range, number of bands per individual, expected (*H*_E_), and observed heterozygosity (*H*_O_) were quantified using the Arlequin version 3.5 [25]. Hardy–Weinberg equilibrium (HWE) and linkage disequilibrium (LD) were performed using GENEPOP 3.4 software [26]. Results of tests for linkage and Hardy–Weinberg disequilibria were corrected for multiple comparisons by applying sequential Bonferroni corrections [18]. Inbreeding coefficient (*F*_IS_) [27] and the significance of these values were calculated for each population with GenePop Web Version 4.0.10 [26,28].

## 4. Conclusions

The wild resource of *M. barbata* has declined in the last decade. Therefore, the development of microsatellites and their application, which can offer an effective tool for understanding genetic variation and population structure in *M. barbata*, is of vital importance. These eight polymorphic microsatellite loci reported here are the first microsatellite markers designed specifically for *M. barbata*, and the versatility of these new primer sets will provide for the further studies on the genetic structure, gene flow, sustainable management and molecular evolution of this susceptible species. The microsatellite loci are sufficient to perceive significant differences among populations of *M. barbata* in different fishing grounds of Asia in future studies.

## Figures and Tables

**Table 1:** The forward (F) and reverse (R) primer sequences, repeat motif, size range and *T*_m_, annealing temperature for eleven microsatellite loci of *Metapenaeopsis barbata*.

Locus	Primer sequence(5′ to 3′)	Repeat motif	Size range (bp)	*T*m °C
MIMB01	F: CAATCGCGCCTCTTACACTT	(CA)_9_	147~161	54
	R: GGCAAAAAGAATGGTAATTGGT			
MIMB02	F: TCTATATGTGTGTCCGCGTGT	(TG)_9_	168~182	54
	R: CATGTTCAGTATGATGTGTCTATCG			
MIMB03	F: AAAACAACGATTTCCAAACAGAA	(TC)_12_	204~218	57
	R: TGAAAATTGCGAATTTCCTTT			
MIMB04	F: TGATTGCGAAGGTCATCAAG	(CT)_15_	180~200	50
	R: TGAAAGGAAAGATTCGAGGAGA			
MIMB05	F: AGTTAACAGCCTCCGGGAACTCC	(AT)_8_	175~193	50
	R: GGACAAGAGGCAGGTACATAG			
MIMB06	F: TTTAATGTGTATTGCGGTCTCC	(GT)_11_	149~155	60
	R: CATACACACACGCAGGACATAG			
MIMB07	F TGCTGGACCTTTGGGTTTATAG	(GT)_10_	240~252	60
	R: CATACAAGCACACGCACAAATA			
MIMB08	F TGGAGGAGATTGGGAGATTG	(GT)_10_	201~205	54
	R: GAATTCGATTGACCGCTTGT			

**Table 2:** Genetic estimates of eight microsatellite loci for *Metapenaeopsis barbata* from Taichung, Taiwan, and Fujian, China.

	Taichung							Fujian						

	*N*_A_	*A*_R_	*H*_O_	*H*_E_	P_HW_	*F*_IS_	*P*_FIS_	*N*_A_	*A*_R_	*H*_O_	*H*_E_	P_HW_	*F*_IS_	*P*_FIS_
MIMB01	3	2.905	0.381	0.431	0.70352	0.118	0.074	3	2.860	0.300	0.303	1.00000	0.010	0.012
MIMB02	3	2.905	0.381	0.431	0.70033	0.118	0.074	3	2.487	0.205	0.303	0.07629	0.325	0.183
MIMB03	2	1.905	0.048	0.048	1.00000	0.000	0.000	3	2.929	0.500	0.482	0.18053	−0.038	0.061
MIMB04	3	3.000	0.286	0.429	0.06301	0.339	0.345	3	2.890	0.324	0.419	0.17474	0.228	0.139
MIMB05	4	4.000	0.474	0.667	0.17390	0.296	0.381	4	3.998	0.538	0.654	0.21398	0.179	0.125
MIMB06	3	3.000	0.476	0.563	0.07654	0.158	0.358	3	2.890	0.324	0.405	0.35083	0.201	0.102
MIMB07	3	3.000	0.333	0.424	0.15270	0.218	0.070	4	3.510	0.229	0.283	0.13777	0.195	0.109
MIMB08	3	3.000	0.238	0.368	0.08563	0.359	0.408	3	2.974	0.225	0.289	0.05880	0.223	0.234
mean	3.00	2.964	0.327	0.420	1.0000	0.226	0.0392	3.25	3.067	0.331	0.392	0.9966	0.158	0.0476

Number of alleles (*N*_A_), allelic richness (*A*r), expected (*H*_E_) and observed (*H*_O_) heterozgosities and significance of deviation from Hardy–Weinberg equilibrium (P_HW_), fixation index (*F*_IS_) and *P*-values of Chi-Square tests for fixation index (*P*_FIS_) for microsatellite loci were estimated.
